# Factors influencing receipt of mental health services for Black women living in Los Angeles County during the perinatal period

**DOI:** 10.1186/s12884-025-08251-2

**Published:** 2025-10-15

**Authors:** Kortney Floyd James, Keren Chen, Sasha Sharon Hindra, Monicque Landell, Kristen R. Choi, Dana C. Beck

**Affiliations:** 1 Department General Internal Medicines, David Geffen School of Medicine , University of California Los Angeles, National Clinician Scholars Program, 1100 Glendon Ave, Suite 900, Los Angeles, CA 90024 USA; 2 Department of Medicine Statistics Core, David Geffen School of Medicine, University of California, Los Angeles, 855 Tiverton Dr, Los Angeles, CA 90024 USA; 3 Sue & Bill Gross School of Nursing, University of California, Irvine, 854 Health Sciences Rd, Irvine, CA 92697 USA; 4 Franklin & Marshall College, 637 College Avenue, Lancaster, PA 17603 USA; 5 School of Nursing Los Angeles, University of California, Los Angeles, 700 Tiverton Dr, Los Angeles, CA 90095 USA; 6 Department of Health Policy and Management, Jonathan and Karin Fielding School of Public Health, University of California, Los Angeles, 650 Charles E Young Dr S, Los Angeles, CA 90095 USA

**Keywords:** Obstetric care, Mental health, Healthcare systems, Access to care, Nursing, Black women

## Abstract

**Background:**

Although California is regarded as a leader in reproductive freedoms and healthcare within the United States, Black women experience persistent inequities in perinatal mental healthcare. Despite healthcare system involvement, a significant proportion of Black women experiencing symptoms of perinatal mood and anxiety disorders (PMADs) go untreated. The purpose of this study was to 1) characterize clinician screening practices for PMADs; 2) describe the extent of perinatal mental health needs; and 3) identify factors within health systems that influence receipt of mental health services among Black women who lived in Los Angeles County, California throughout the perinatal period.

**Methods:**

Secondary analysis of cross-sectional data from the Black Mothers’ Mental Wellness Study. The sample consisted of 110 Black women who self-reported either experiencing PMAD symptoms or being recently diagnosed with a PMAD. Results: Most of the sample received a clinical assessment for depression during pregnancy (78.2%, *n*=86) or after childbirth (70.0%, *n*=77). Among those who received a depression assessment, a nurse most often performed the screening. Among the 102 participants who experienced or were diagnosed with a PMAD throughout the perinatal period, 80 (78.4%) received mental health services, including counseling/therapy, support groups, or prescribed medication. Black women who received education about postpartum depression during pregnancy or after childbirth (OR=5.1, CI=-1.64-11.85) had almost 5 times higher odds of receiving mental health treatment than those who had not.

**Conclusions:**

The findings from this analysis emphasize a gap in standard obstetric practice, which may result in undetected and untreated PMAD symptoms. Findings also highlighted the role of nurses in obstetric care, as well as the importance of anticipatory guidance regarding PMADs for Black women in Los Angeles County.

Black women who experience symptoms of perinatal mood and anxiety disorders (PMADs) may be at risk for suicide, but their symptoms are often undetected and/or untreated by clinicians within health systems [1]. Although California is regarded as a leader in reproductive freedoms and healthcare within the United States (US), Black women experience persistent inequities in perinatal mental healthcare. In Los Angeles County (LAC), the most populous county in the US, Black women make up 9% of the population but have the highest prevalence of experiencing PMAD symptoms (38%) in comparison to women of other racial groups [2]. Further, among Black women in California with moderate to severe PMAD symptoms during the prenatal or postpartum period, only 13.5% − 30.9% receive treatment [3, 4]. If Black women experiencing PMAD symptoms are not identified or provided adequate support/referrals, they may present to the emergency department with more severe PMAD symptoms. Studies from San Francisco suggest that Black women experience frustration with their inability to access outpatient mental health services for PMADs [5]. Although Black women experiencing PMAD symptoms may quickly receive acute treatment in the emergency department for suicidal ideation, including hospital admission [1, 6], emergency and inpatient care utilization is not optimal when preventive services, early treatment, and long-term support could prevent these expensive and disruptive forms of treatment. When obstetric clinicians screen for PMADs and refer patients for mental health services, mental health outcomes improve, and appropriate mental health service utilization increases [7]. Therefore, it is critical that Black women experiencing PMAD symptoms are identified, referred to, and receive mental health services early.

While a myriad of factors influences PMAD symptom detection, diagnosis, and receipt of mental health services among Black women [8–10], few studies evaluate health system factors that lead to various types of mental health treatment. A study from a large healthcare system in Minnesota [11], examined factors related to the receipt of PMAD screening and found that both clinician type and insurance status influenced screening rates. Specifically, patients of family medicine physicians and obstetricians were more likely to receive prenatal PMAD assessments in comparison to patients of certified nurse midwives and nurse practitioners. The study also found that women who were Black, American Indian, multi-racial, or Hispanic and had Medicaid/Medicare were less likely to receive postpartum PMAD screening, suggesting potential racial and insurance-based disparities in screening practices. Additionally, among all clinician types, obstetricians were the most likely to provide postpartum PMAD screening. However, a study of Black women in California [4] found that clinician type did not significantly influence screening rates, suggesting that other structural or systemic factors may be driving disparities in PMAD screening. Floyd James and colleagues also found that Black women with employer-sponsored/private insurance were more likely to receive mental health services compared to those with public insurance, further highlighting the role of insurance type in access to care. Given these mixed findings, this study seeks to expand knowledge by examining screening trends and contributing factors specific to LAC.

There is a need for research to meet gaps in knowledge about key health system factors that affect disparities in perinatal mental healthcare delivery. Identifying health system factors implicated in mental treatment might improve our understanding of modifiable intervention targets that can reach a large population of Black women and reduce observed disparities. The purpose of this study was to: (1) characterize clinician PMAD screening practices; (2) describe the extent of perinatal mental health needs; and (3) identify factors within health systems that influence receipt of mental health services among Black women in the postpartum period living in LAC.

## Methods

### Design

This study was a secondary analysis of the Black Mothers’ Mental Wellness Study [12], a web-based survey that used a cross-sectional design to examine the relationships between racism and perinatal mental health among Black women living in LAC. In this analysis, we analyzed data from the web-based survey that captured participants’ mental health history during pregnancy and after childbirth, and receipt of mental health services during the same time points. Survey responses were collected between August 2022 and April 2023. The original study was conducted in accordance with prevailing ethical principles and Institutional Review Board approval was obtained from University of California, Los Angeles. Details regarding the original study are available elsewhere [12].

### Sample

The convenience sample of the original study was recruited throughout LAC using digital and printed flyers on social media platforms and locations that serve Black women, including obstetric and pediatric clinics, as well as health and community organizations. Individuals were eligible to participate in the study if they (1) self-identified as Black women (2), were 18 years of age or older (3), lived in LAC (4), were 6 weeks to 12 months postpartum, and (5) had a singleton birth. The Black Mothers’ Mental Wellness Study had a total sample of 231 Black postpartum women. The analytical sample for this study was limited to any participants who self-reported a history of a PMAD, recent diagnosis of a PMAD (either during or after most recent pregnancy), or feeling as if they had a PMAD, regardless of clinician diagnosis. These inclusion criteria resulted in an analytical sample of 110 Black postpartum women.

### Measures

#### Outcome

##### Receipt of mental health services

The survey contained four items to assess whether participants had taken prescribed medications or utilized therapeutic mental health services for their PMAD symptoms during pregnancy and/or after childbirth. The initial item asked, “At any time in your life, have you taken medicine prescribed by a healthcare provider (such as a physician, physician assistant, nurse practitioner, or physician assistant) for a mental health condition(s)?” There were then two follow-up questions presented to participants who indicated that they had taken prescribed medication: (1) Please select the mental health condition(s) that the medicine was prescribed for while you were pregnant (2), Please select the mental health condition(s) that the medicine was prescribed for after you gave birth to your baby.” Participants had the following multiple-choice response options: anxiety, bipolar, depression, postpartum depression, and schizophrenia. The survey contained one item to assess participants’ utilization of therapeutic mental health services, “At any time in your life, have you gone to therapy, counseling, or support groups (including 12-step programs) for the mental health condition(s) you listed? Even if a few times.” Response options were: (1) yes; (2) no, because I was not interested; (3) no, because I could not afford therapy/counseling; (4) no, because I could not find a therapist/counselor/support group; (5) no, for some other reason.

#### Exposures

The primary exposures were factors within the healthcare system, including receipt of PMAD education (no/yes), type of clinician who provided PMAD education (advanced practice clinician, doula, midwife, nurse, physician), type of health insurance (employer-sponsored, Medi-Cal, none, other), receipt of prenatal PMAD screening (no/yes), and receipt of postpartum PMAD screening (no/yes).

#### Sociodemographics

Participant sociodemographics included the following characteristics: age (continuous), age of infant (continuous), birth type (cesarean, vaginal), healthcare insurance (employer-sponsored, Medi-Cal, other government-sponsored insurance, self-pay), history of mental health condition prior to pregnancy (no/yes), and monthly income (less than $1,000; $1,000–$1,999; $2,000-$2,999; $3,000–$3,999; $4,000 to $4,999; $5,000 or more). Each sociodemographic characteristic was assessed via participant self-report in the Qualtrics^®^ survey.

### Data analysis plan

Sociodemographic characteristics including age, income, education, employment status, insurance status, relationship status, number of children, infant age, birth type were analyzed using descriptive statistics for the entire data sample and between groups that received or did not receive mental health services. Bivariate difference were compared between these two groups using t-test for continuous variables and Fisher’s exact test for categorical variables. Contingency tables and chi-square test were used to compare the receipt of perinatal depression screening frequencies and mental healthcare receipt by mental health history. A logistic regression model was estimated to identify factors that influence the receipt of mental health services among Black women living in LAC. The binary dependent variable in the logistic regression model was recoded based on the binary variables of the receipt of mental health prescriptions and the receipt of mental health treatment – an individual was considered to have received mental health services if he/she had received either mental health prescriptions or any form of mental health treatment. Variables in the logistic regression model were selected known and hypothesized association from literature including screening for depression during or after pregnancy, having a doula during pregnancy, education about postpartum depression and anxiety during pregnancy and after childbirth, age, and income. All statistical tests were carried out using R Statistical Software (v4.2.3; R Core Team 2023) with a two-tailed statistical significance set at *p* < 0.05 and a 95% confidence interval.

## Results

On average, the participants were 31 years old (SD 3.8), 7.1 months postpartum (SD 5.5), and had two children. Most of the sample earned a college degree (77.3%, *n* = 85), had employer-sponsored/private insurance (38.2%, *n* = 42), and was married or living with their partner (85.5%, *n* = 94). The distribution of sample characteristics for the analytic sample of participants with a history of mental health conditions or a recent experience with PMAD can be found in Table 1. Most women in the sample received a clinical assessment for depression during pregnancy (*n* = 86, 78.2%) or after childbirth (*n* = 77, 70.0%), yet there were still 12.7% (*n* = 14) of women in the sample who had never received any such assessment (Table 2). Among those who received a depression assessment, a nurse most often performed the screening during pregnancy (*n* = 67, 60.9%) and after childbirth (*n* = 57, 51.8%). Among the 102 participants who thought they had or were told they had mental health conditions while pregnant or after birth, 80 (78.4%) received services, including counseling/therapy, participated in support groups, or took prescribed medication (Table 3). The odds of receiving mental health services for Black women who received education about postpartum depression during pregnancy or after childbirth were almost 5 times higher than those who had not (OR = 5.1, 95% CI=−1.64–11.85) (Table 4).

**Table 1:** Sociodemographic characteristics of the sample (*N* = 110)

^1^Sample Characteristic	Total sample (*N* = 110) *N*, % (Mean ± SD)	Received mental health services (*N* = 85) *N*, % (Mean ± SD)	Did not receive mental health services (*N* = 25) *N*, % (Mean ± SD)	*p* value
Age (year)	31.3 ± 3.8	31.3 ± 4.0	31.5 ± 3.2	0.796
Infant age (month)	7.1 ± 5.5	7.5 ± 6.1	6.1 ± 2.7	0.112
Number of children given birth to	1.8 ± 0.9	1.9 ± 0.9	1.3 ± 0.5	< 0.001
Birth type				0.999
Vaginal birth	32, 29.1%	25, 29.4%	7, 28.0%	
Cesarean (C-section) birth	78, 70.9%	60, 70.6%	18, 72.0%	
Employment status				0.701
On maternity leave	19, 17.3%	14, 16.5%	5, 20.0%	
Unemployed	15, 13.6%	12, 14.1%	4, 16.0%	
Self-employed	8, 7.3%	5, 5.9%	3, 12.0%	
Part-time/occasional	28, 25.5%	22, 25.9%	6, 24.0%	
Full-time	40, 36.4%	32, 37.7%	7, 28.0%	
Insurance				0.599
Medi-Cal/Medicare	27, 24.6%	21, 24.7%	6, 24.0%	
Other government insurance	4, 3.6%	3, 3.5%	1, 4.0%	
Employer-sponsored insurance	42, 38.2%	35, 41.2%	7, 28.0%	
Covered California	23, 20.9%	17, 20.0%	6, 24.0%	
No Insurance	14, 12.7%	9, 10.6%	5, 20.0%	
Education				0.439
Did not complete high school	2, 1.8%	2, 2.4%	0, 0.0%	
GED	2, 1.8%	2, 2.4%	0, 0.0%	
High school diploma	21, 19.1%	15, 17.7%	6, 24.0%	
Associate degree	29, 26.4%	19, 22.4%	10, 40.0%	
Bachelor’s degree	44, 40.0%	37, 43.5%	7, 28.0%	
Master’s degree	9, 8.2%	8, 9.4%	1, 4.0%	
Doctorate Degree	3, 2.7%	2, 2.4%	1, 4.0%	
Income				0.501
Less than $1,000	13, 11.8%	10, 11.8%	3, 12.0%	
$1,000 to $1,999	30, 27.3%	22, 25.9%	8, 32.0%	
$2,000 to $2,999	22, 20.0%	21, 24.7%	2, 8.0%	
$3,000 to $3,999	24, 21.8%	15, 17.7%	8, 32.0%	
$4,000 to $4,999	10, 9.1%	8, 9.4%	2, 8.0%	
$5,000 or more	11, 10.0%	9, 10.6%	2, 8.0%	
Relationship status				0.837
Single/Never married	8, 7.3%	6, 7.1%	1, 4.0%	
Married/Living with a partner	94, 85.5%	72, 84.7%	23, 92.0%	
Divorced/Separated	7, 6.4%	6, 7.1%	1, 4.0%	
Widowed	1, 0.9%	1, 1.2%	0, 0.0%	

^1^Analytic sample is limited to participants with a history of mental health conditions or a recent experience of PMAD either during or after the most recent pregnancy (whether self-reported or diagnosed). Analytic *N* = 110

**Table 2:** Comparison of receipt of perinatal depression screening frequencies

		Postpartum Screen		
		NO	YES	Margin
Prenatal Screen	NO	14 (12.7%)	10 (9.1%)	24 (21.8%)
	YES	19 (17.3%)	67 (60.9%)	86 (78.2%)
	Margin	33 (30.0%)	77 (70.0%)	110 (100%)

Chi-square test *p* = 0.001

**Table 3:** Comparison of mental health treatment receipt by mental health history

		Received mental health care		
		NO	YES	Margin
Had mental health conditions prenatally or postpartum	NO	3 (37.5%)	5 (62.5%)	8 (100%)
	YES	22 (21.6%)	80 (78.4%)	102 (100%)
	Margin	25	85	110

Fisher’s exact test *p* = 0.378

**Table 4:** Factors associated with mental health services receipt for Black women throughout the perinatal period (*N* = 110)

^1^Outcome	Variable	OR	SE	95% CI		*p*-value
				LL	UP	
^2^Mental health services	Screened for depression during pregnancy or after childbirth	4.03	2.99	−1.84	9.90	0.060
	Doula care during pregnancy	0.97	0.58	−0.18	2.11	0.957
	Received education about depression during pregnancy or after childbirth	5.11	3.44	−1.64	11.85	0.015
	Received education about anxiety during pregnancy or after childbirth	0.57	0.42	−0.25	1.40	0.447
	Birth type (C-section)	1.01	0.62	−0.21	2.23	0.989
	Income, per category	0.89	0.19	0.53	1.26	0.588
	^3^Insurance, Other government insurance	0.64	0.91	−1.15	2.42	0.751
	^3^Insurance, Employer-sponsored insurance	1.35	1.03	−0.67	3.37	0.695
	^3^Insurance, Covered California	0.71	0.58	−0.42	1.84	0.673
	^3^Insurance, No Insurance	0.41	0.36	−0.29	1.10	0.303

95% CI = 95% Confidence Interval

^1^Analytic sample of the logistic regression model is limited to participants with a history of mental health conditions or a recent experience of PMAD either during or after the most recent pregnancy (whether self-reported or diagnosed). Degree of Freedom = 109

^2^Binary dependent variable: At any time in your life, have you taken medicine prescribed by a healthcare provider (such as a physician, physician assistant, nurse practitioner, physician assistant) for a mental health condition(s), and/or at any time in your life, have you gone to therapy, counseling, or support groups (including 12-step programs) for the mental health condition(s) you listed?

^3^Reference category: Medi-Cal/Medicare

## Discussion

This study sought to characterize clinician PMAD screening practices, describe the extent of perinatal mental health needs, and identify factors within health systems that influence the receipt of mental healthcare services among Black women in the postpartum period who lived in LAC.

### Clinician screening practices for perinatal mood and anxiety disorders

Almost 13% of the women in this study were not screened for PMADs at any point during the perinatal period. While any missed PMAD screening is concerning, the missed screening rate for the women in this study is low in comparison to previous studies. Another study of Black women living in California who had given birth in 2016 [4] found that 21% were not screened for PMADs during the postpartum period. Further, a retrospective study conducted in Chicago, Illinois in 2019, which included a medical record review found that 69% and 78% of Black women did not receive PMAD screening during the prenatal or postpartum period, respectively [13]. It is possible that the clinical policies and procedures used in CA and LAC are methodical, ensuring that the majority of pregnant and postpartum individuals are screened for PMADs. However, the studies conducted in CA relied on participants’ self-report and recall memory while the study in Chicago utilized medical record data. It is possible that the participants further along in the postpartum period may be less likely to have an accurate recollection of what occurred during earlier visits after childbirth [14]. However, clinicians may record the details of patient visits during or soon after the encounter [15] and therefore may be more accurate regarding screening rates. A recent study linked single state Medicaid data with Pregnancy Risk Assessment and Monitoring system data to understand gaps in patient self-report of PMAD symptoms and corresponding clinical diagnosis. This study found that white women had three times the odds of a PMAD diagnosis if they reported symptoms compared to Black women [16], suggesting that disparities in diagnosis may be influenced by multiple factors, including racial bias, differences in insurance coverage, clinician training and knowledge, and systemic barriers to care.

Among the study participants who received a clinical assessment for PMADs at any time, a nurse most often performed the assessment. Within healthcare systems, nurses often provide the PMAD screening tool(s) to patients but the lead clinician (i.e., physician, midwife) reviews and discusses the results with patients, and provides referrals or treatment, as needed [17]. Nurses are often utilized in maternal, infant, and early child home visiting programs (MIECHV), such as the Nurse-Family Partnership, to conduct PMAD screening/detection, diagnosis, and referral [18, 19]. These nurse led MIECHV programs have demonstrated PMAD screening and treatment rates much higher than those within healthcare systems. Tandon and colleagues [20] examined the PMAD screening and treatment rates of 13 MIECHV programs in eight states and determined that more than 96% of clients were screened for PMADs; of clients with positive screenings, almost 70% received mental health treatment. These MIECHV programs reflect the importance of specialized education and training to support nurses’ ability to screen women for PMADs and provide effective education to support women’s receipt of perinatal mental health treatment [18, 19].

Our findings further reinforce the critical link between screening and treatment, showing that individuals with a history of mental health conditions or recent experience of PMADs were four times higher odds to receive mental health services when screened for depression during pregnancy or postpartum. While this result approached statistical significance (*p* = 0.06), this finding aligns with existing evidence suggesting that systematic screening efforts can improve access to mental health care and facilitate timely intervention for perinatal individuals experiencing mental health concerns [21–23].To improve PMAD screening rates and reduce incidences of undiagnosed and untreated PMADs, future research could utilize randomized control trials to examine the efficacy and appropriateness of nurse-led MIECHV models of care within healthcare systems to address the mental health needs of Black pregnant and postpartum women.

### Extent of perinatal mental health needs for Black women

Among the participants who either felt they had or were told they had a mental health condition throughout the perinatal period, almost 22% did not receive mental health treatment (i.e., therapy, counseling, support group, medication). This reflects a gap in the linkage between diagnosis and treatment and is congruent with prior research that indicates Black women are less likely to receive early recognition and treatment for mental health conditions and more likely to receive emergency services or inpatient hospitalization [24]. The sample in this study represents a group with socially prescribed advantages (e.g., married, college educated, commercial health insurance) traditionally thought of as ‘protective’ factors in relationship to perinatal mental health [25]. Therefore, we might expect the magnitude of need in relation to receipt of mental health treatment to be greater among Black women in LAC with less socially prescribed advantages, although this has not been documented in the scientific literature. This underscores the pressing need for targeted interventions and support systems to bridge the gap between Black women experiencing PMAD symptoms and receiving mental health treatment to ensure that those in need of services receive timely and effective mental health care.

### Factors that influence receipt of perinatal mental healthcare services

Although the Black women in this study who received anticipatory guidance about PMADs had higher odds of receiving mental health treatment than those who did not, previous research findings have illustrated that anticipatory guidance alone may not be enough to facilitate Black women’s receipt of mental health care services. Bingham and colleagues [26] suggest that providing prenatal education regarding the signs and symptoms of PMADs may reduce postpartum morbidity or mortality caused by untreated PMAD symptoms. The findings of this current study align with Bingham and colleagues’ conclusion, further emphasizing the critical role of prenatal education in improving self-identification of PMAD symptoms and promoting care-seeking behavior within this population. However, strategies beyond awareness-raising are needed to address disparities in access to mental health care. Qualitative research [27, 28] has shown that despite being aware of PMAD symptoms, Black women often do not recieve mental health services. Instead, many rely on family members, support groups, or self-help interventions. Thus, a multi-faceted approach is needed within healthcare systems to reimagine perinatal mental health care for Black women [29]. The approach would include policies to ensure that all healthcare staff who interact with pregnant and postpartum women are prepared to holistically assess and address the various structural determinants of health that negatively impact Black women’s mental health, and act as barriers to receiving comprehensive, culturally appropriate mental health treatment.

## Limitations

There are some limitations of this study inherent to the sampling technique, setting, and study design. Convenience sampling may have led to selection bias, such that women who were knowledgeable about mental health may be more likely to advocate for themselves, resulting in the higher rates of screening/receipt of mental health treatment than is typical. Further, convenience sampling may have recruited women who were either more motivated to participate in the study, or simply those able to recall being screened for PMADs. Although the sample size was powered to detect statistically significant differences in mental health treatment receipt, the small sample size within one region limits the generalizability of the findings. Futher, while this study intentionally focuses on Black women throughout the perinatal period due to well-documented disparities, a key limitation is the lack of inclusion of other racial and ethnic groups, restricting our ability to fully examine disparities in screening and treatment across diverse populationsDue to the cross-sectional design of the Black Mothers’ Wellness Study, causal relationships between the variables cannot be determined. Additionally, this study utilized self-reporting, which may be subject to recall bias, thereby impacting the accuracy of participants’ responses. Lastly, the quantitative nature of the analysis does not allow participants to elaborate on their experiences in seeking mental health treatment, thereby vital context regarding barriers to receipt of mental health treatment is missing from this analysis. Future qualitative research could provide further insight into the barriers to treatment receipt within LAC but also describe the various non-medical pathways to healing Black women utilize when experiencing PMADs.

## Conclusion

This analysis delineated the practices employed by clinicians when screening for PMADs, described the magnitude of perinatal mental health needs, and discerned the systemic factors that influence the receipt of perinatal mental healthcare services for Black women residing in LAC. Our study found that 12% of Black women in the study were not screened for PMADs during the perinatal period, a rate much lower than reported in previous research, but a gap in standard clinical practice nonetheless, which may result in undetected PMAD symptoms, potentially leading to more severe manifestations. The findings from this study shed light on the critical issue of PMAD detection, diagnosis, and treatment among Black women in LAC. Lastly, findings suggest that future research should explore the potential efficacy and suitability of integrating nurse led PMAD screening, diagnosis, and referral models of care within health systems to address the mental health needs of Black women.

## Data Availability

The datasets used and/or analyzed during the current study are available from the corresponding author on reasonable request.
